# *Culex quinquefasciatus* Storage Proteins

**DOI:** 10.1371/journal.pone.0077664

**Published:** 2013-10-29

**Authors:** Larissa A. Martins, Andréa C. Fogaça, A. Tania Bijovsky, Rebeca Carballar-Lejarazú, Osvaldo Marinotti, André F. Cardoso

**Affiliations:** 1 Departamento de Parasitologia, Instituto de Ciências Biomédicas, Universidade de São Paulo, São Paulo, SP, Brasil; 2 Department of Molecular Biology and Biochemistry, University of California Irvine, Irvine, California, United States of America; New Mexico State University, United States of America

## Abstract

Insect storage proteins accumulate at high levels during larval development of holometabolous insects. During metamorphosis they are degraded, supplying energy and amino acids for the completion of adult development. The genome of *Culex quinquefasciatus* contains eleven storage protein-coding genes. Their transcripts are more abundant in larvae than in pupae and in adults. In fact, only four of these genes are transcribed in adults, two of which in blood-fed adult females but not in adult males. Transcripts corresponding to all *Cx. quinquefasciatus* storage proteins were detected by RT-PCR, while mass spectrometric analysis of larval and pupal proteins identified all storage proteins with the exception of one encoded by Cq LSP1.8. Our results indicate that the identified *Cx. quinquefasciatus* storage protein-coding genes are candidates for identifying regulatory sequences for the development of molecular tools for vector control.

## Introduction

Insect storage proteins are members of a large arthropod protein family, which includes haemocyanins, responsible for oxygen transport, as well as pro-phenoloxidases and dipteran hexamerin receptors [1]–[3]. Storage proteins, also known as larval serum proteins (LSP) or hexamerins, accumulate at high levels during the late larval stage in holometabolous insects, reaching in some cases 60% of all soluble proteins of the organism [4]. The accumulated storage proteins constitute a main source of amino acids and energy for metamorphosis [5]. The functions of some storage proteins however extend beyond their participation in metamorphosis. They also are believed to serve as carriers of hormones, components of the cuticle and to participate in immune defense [2]. Storage proteins also have been detected in developing ovaries and testes, thus suggesting a role in gonad maturation [6], [7]. Circumstantial evidence that hexamerins are targeted for egg production has also been obtained [8]–[11].

The mosquito *Culex quinquefasciatus* is a significant vector of the filarial nematode, *Wuchereria bancrofti*, and can transmit under favorable conditions a variety of encephalitis-causing viruses, including West Nile Virus [12]–[14]. It also is a significant nuisance pest to humans, having a persistent and nocturnal biting behavior [15], [16]. Patients allergic to mosquito bites may present severe reactions [17], [18]. It is therefore relevant to identify and characterize genes that may be used to develop genetic control strategies for this mosquito and its associated pathogens. Towards this goal, transgenesis technologies were established for this insect [19] and a whole-genome sequencing project has been conducted [20], [21] (http://cpipiens.vectorbase.org/index.php).

Here we describe our efforts to identify the *Culex quinquefasciatus* storage proteins and describe their corresponding protein and transcript accumulation patterns. The data are consistent with the expression profiles of the storage protein encoding genes of the yellow fever mosquito, *Aedes aegypti*, and the human malaria vector, *Anopheles gambiae* [22], [23]. In addition, our results support the conclusion that these genes are candidates for donating regulatory sequences for driving transgene expression in transgenic mosquitoes and for developing genetic tools for controlling disease transmission.

## Materials and Methods

### Ethics Statement

The protocols used in this work were approved by the Animal Experimentation Ethics Committee of the Institute of Biomedical Sciences (University of São Paulo, São Paulo, Brazil – process number CEAU 103).

### Animals

*Culex quinquefasciatus* (PIN strain - [24]) were raised at 26°C, 70–80% relative humidity and under a photoperiod of 12 h dark-12 h light. Adults were fed 10% sucrose solution *ad libitum*. When necessary, 5–7 day-old adult females were fed on Balb/c mice anaesthetized with 0.3 mg/kg of xylazine hydrochloride (Calmiun, Agner União, Brazil) plus 30 µg/kg of acepromazine (Acepran, Univet SA, Brazil).

Blood-fed mosquitoes were allowed to lay eggs in a small bowl of water placed inside the cage. Larvae were raised in plastic trays containing 450 ml of distilled water and fed with fish food (Sera® Vipan, Heinsberg, Germany). Sexing of *Cx*. *quinquefasciatus* pupae was based on separation by size, larger pupae were considered females [25]. Adults were kept in cages with access to a 10% sucrose solution *ad libitum*.

### Dissection and Preparation of Fat Body Extracts

Larval fat bodies were dissected, homogenized in 10% trichloroacetic acid and the precipitated proteins were collected by centrifugation at 8,500×g for 10 min. The pellet was washed once with 100% acetone and washed again with ether: chloroform (1∶1, v/v). The resulting pellet was dried. Alternatively, fat bodies of pupae and adults were dissected and homogenized in PBS (phosphate buffered saline −137 mM NaCl, 3 mM KCl, 7 mM Na_2_HPO_4_, 1 mM KH_2_PO_4_) pH 7.2 containing 1 µl/ml of a cocktail of protease inhibitors (10 µM leupeptin, 1 µM pepstatin, 10 µM chymostatin, 10 µM antipain, 5 µM PMSF and 5 µM/ml E-64) as described by Cardoso et al [26]. Protein content of samples was determined using the Bradford assay [27] (Bio-Rad Laboratories, Brazil) according to the manufacturer’s instructions.

### Hemolymph Extraction

Around 0.5 µl of PBS pH 7.2 containing the protease inhibitors described above were injected into pupae or adults. The drop expelled from the cephalothorax (pupae) or leg injury (adults) was collected with a microcapillary pipette and stored at −20°C until analysis by SDS-PAGE.

### Protein Electrophoresis

Fat body extracts and hemolymph samples were mixed with an equal volume of sample buffer (62 mM Tris, 50 mM DTT, 0.2% SDS, 10% glycerol, and 0.01% bromophenol blue) and heated at 100°C for 2 min. Proteins were resolved in an 8% gel by SDS-PAGE [28] and visualized by staining with 0.2% Coomassie Brilliant Blue R250 (w/v) dissolved in ethanol, acetic acid, water (45∶10:45, v/v/v) (Bio-Rad Laboratories, Brazil). Molecular masses were estimated using the following standards: myosin (202 kDa), β-galactosidase (116 kDa), bovine serum albumin (98 kDa), and ovalbumin (47 kDa) (Bio-Rad Laboratories, Brazil).

### Mass Spectrometry Analyses

Protein bands were excised from the gel and in-gel digested as previously described by Shevchenko et al. [29]. Whole pupae preparations were homogeneized in trypsin-digest buffer as described [30]. After desalting using C_18_ reverse phase tips (Zip Tip, Millipore), tryptic fragments were analyzed by nano-HPLC- ESI-MS/MS using the same equipment and procedure described [31], [32].

MS/MS analyses were performed with Bioworks Browser version 3.3 (Thermo Scientific, USA) and peptides were identified with the Sequest® algorithm using a NCBI non-redundant database of *Culex*. Alternatively, for data acquisition, LC-MS was carried out using a Waters ACQUITY UPLC® system coupled to a SYNAPT®G2 mass spectrometer. Masslynx 3.5 software (Waters Corporation, Milford, MA, USA) was used for data acquisition, processing, and determination of peptide sequences. The protein identification was performed with the ProteinLynx Global Server Web (Waters) with SwissProt database for *Cx. quinquefasciatus*. Peptides were validated using protein probability ≤1×10^−7^, dCN ≥0.05, and Xcorr of 1.5, 2.2, and 2.7 for singly, doubly, and triply charged peptides, respectively. Only proteins represented by two or more validated peptides were considered.

### Oligonucleotide Design

Primers for RT-PCR amplification, detection and quantification of storage protein-coding transcripts were designed using the Primer3 program [33] and the transcript sequences available at VectorBase [21] as template (Table S1). Primers were synthesized by Life Technologies (USA).

### RNA Extraction

Total RNA was extracted from whole fourth instar larvae, early and late female pupae (about 12 and 36 hours after pupation respectively), male pupae (about 36 hours after pupation), adult females 1, 3, and 5 days after emegence, 7 day-old females 24 hours after a blood meal, and adult males 7 days after emegence using Trizol® (Invitrogen, Brazil). Residual genomic DNA was removed by incubation with DNase I, Amp Grade (Invitrogen, Brazil) and RNA integrity was evaluated by agarose gel electrophoresis. RNA was quantified on a NanoDrop ND-1000 spectrophotometer (Thermo Scientific, USA).

### RT-PCR

Reverse transcription (RT) was carried out with 2.2 µg of total RNA, 500 ng of oligo dT (Invitrogen, Brazil) and the SuperScript II first-strand synthesis system (Invitrogen, Brazil). PCR was conducted with 100 ng of cDNA as template and 500 nM of each primer (Table S1). Reactions were performed in an Eppendorf ® thermocycler using the thermal program of 15 min at 95°C, followed by 25–30 cycles of 95°C for 15 s, 55°C for 30 s, and 72°C for 30 s. Amplicons were resolved on 1.5% agarose gels, stained with Gel Red® (Uniscience, Brazil) and visualized on an Eagle Eye video system (Stratagene, USA).

### Quantitative RT-PCR

Quantitative RT*-*PCR (qRT-PCR) was performed using a Mastercycler® ep realplex 2 apparatus (Eppendorf) in 96-well optical reaction plates (ABgene, USA). One hundred ng of cDNA template, 500 nM of each primer (Table S1), and 10 µl of SYBR green QuantiMix (Biotools, USA) were used for each reaction with final volume of 20 µl. The thermal cycling program was: 15 min at 95°C, followed by 40 cycles at 95°C for 15 s, 55°C for 30 s, and 72°C for 30 s. Amplification efficiency for all primer pairs was determined as greater than 90%. The ribosomal protein 49 gene (rp49, CPIJ001220) was used as reference, constitutively expressed gene. The relative accumulation of transcripts encoding storage proteins was calculated using the 2^−ΔΔC^T method [34] and the 5 d after emergence adult female sample as reference condition. Data corresponded to three independent biological samples. All samples were analyzed in triplicate.

### Sequencing and Analysis

Genomic DNA and cDNA for all the genes were amplified and sequenced on both strands with BigDye Terminator v3.1 (Applied Biosystems, Brazil) on an automatic sequencer model ABI 3100 (Applied Biosystems, Brazil). Resulting sequences were compared with those in the major databases GenBank and VectorBase. Alignment was performed using the BioEdit [35] and Clustalw programs [36]. Signal peptides were predicted using the SignalP 4.0 algorithm [37].

## Results

### *In silico* Identification of *Cx. quinquefasciatus* Storage Protein-coding Genes

Previously described *Aedes aegypti, Anopheles gambiae* and *Drosophila melanogaster* storage protein sequences were used as queries to search for similar proteins in the *Culex quinquefasciatus* protein database [21]. Next, tBLASTn searches were run against the assembled *Culex quinquefasciatus* genome to search for non-annotated or miss-annotated loci encoding storage proteins. Our searches resulted in a set of eleven genes putatively encoding insect storage proteins (Table 1). Eight of the genes encode proteins similar to the *D. melanogaster* LSP1 subunits and were accordingly named Cq LSP 1.1 to Cq LSP 1.8. Sequences similar to *D. melanogaster* LSP1 alpha, beta and gamma subunits were identified in *Cx*. *quinquefasciatus*. The other three are more similar to the *D. melanogaster* LSP2 polypeptide and were named Cq LSP 2.1 to Cq LSP 2.3. All genes encode proteins with amino terminal signal peptides, indicative of their secretory nature. The calculated molecular masses of the mature peptides range between 76 and 83 kDa.

**Table 1:** Physicochemical properties *Cx. quinquefasciatus* storage proteins and their similarities with proteins of other insects.

	Gene/Protein ID	number ofAA residues	Molecularmass matureprotein	pI mature protein	% TYR+PHE	% MET	Potential N-glycosylation sites	*Aedes aegypti* best match	*Anopheles darlingi* best match	*Anopheles gambiae* best match	*Drosophila melanogaster* best match
**LSP1-like**	CPIJ009032 **Cq LSP 1.1**	666	78759	5.8	**16.82** (8.71+8.11)	4.35	**179 NFTS** 322 NGTI 479 NYTL	AAEL013990	ADAR006325	AGAP010658	FBgn0002562 Lsp1alpha
	CPIJ009033 **Cq LSP 1.2**	667	80428	6.1	**22.48** (11.99+10.49)	3.75	**186 NYTA**	AAEL013981	ADAR007061	AGAP010657	FBgn0002563 Lsp1beta
	CPIJ009506 **Cq LSP 1.3**	682	83003	6.1	**15.83** (8.65+7.18)	**14.08**	**189 NYTQ** 374 NSTF	AAEL008045	ADAR000447	AGAP005768	FBgn0002563 Lsp1beta
	CPIJ018824 **Cq LSP 1.4**	675	80952	6.12	**20.15** (10.22+9.93)	4.3	85 NLTY **187 NYTA**	AAEL000765	ADAR007061	AGAP010657	FBgn0002563 Lsp1beta
	CPIJ018825 **Cq LSP 1.5**	663	79340	6.56	**19.76** (10.11+9.65)	4.07	85 NLTY **187 NYTA**	AAEL000765	ADAR007061	AGAP010657	FBgn0002563 Lsp1beta
	CPIJ006537 **Cq LSP 1.6**	675	80968	5.95	**20.15** (10.22+9.93)	4.3	85 NLTY **187 NYTA** 672 NETY	AAEL000765	ADAR007061	AGAP010657	FBgn0002563 Lsp1beta
	CPIJ006538 **Cq LSP 1.7**	675	80951	6.12	**20.14** (10.07+10.07)	4.3	85 NLTY **187 NYTA**	AAEL000765	ADAR007061	AGAP010657	FBgn0002563 Lsp1beta
	CPIJ000056 **Cq LSP 1.8**	630	76275	7.04	**20.16** (10.00+10.16)	3.02	**184 NYTL**	AAEL008045	ADAR000447	AGAP005768	FBgn0002564 Lsp1gamma
**Lsp2-like**	CPIJ007783 **Cq LSP 2.1**	690	82153	5.63	**18.04** (11.10+7.25)	2.46	5 NTSQ **186 NYTG** 334 NFTV	AAEL008817	ADAR009474	AGAP001345	FBgn0002565 Lsp2
	CPIJ001820 **Cq LSP 2.2**	690	82240	5.76	**16.66** (10.14+6.52)	3.19	**181 NYTG**	AAEL013759	ADAR008011	AGAP001657	FBgn0002565 Lsp2
	CPIJ001822 Cq **LSP 2.3**	676	80285	6.0	**18.04** (9.76+8.28)	3.85	**184 NYTG** 331 NITT	AAEL013757	ADAR004610	AGAP001659	FBgn0002565 Lsp2

The molecular masses, isoelectric points and amino acid compositions were calculated using Protean (DNASTAR, Madison, WI, USA). Predictions of glycosylation sites were conducted at NetNGlyc 1.0 Server [58]. Similarities were determined by blastp searches against NCBI and Vectorbase protein databases.

The amino acid composition of the *Cx. quinquefasciatus* storage proteins indicate elevated contents of tyrosine and phenylalanine (Tyr+Phe ranges from 16 to 22% of the total amino acids). One of them, Cq LSP 1.3 also contains a high content of methionine (14%) (Table 1 and Table S2). All of the predicted peptides contains at least one putative N-glycosylation sites. One of the predicted N-glycosylation sites is conserved in all eleven *Cx. quinquefasciatus* storage proteins (eg position 184 (NYTG) in Cq LSP 2.3 and 186 (NYTA) in Cq LSP 1.2, Table 1).

**Figure 1:**
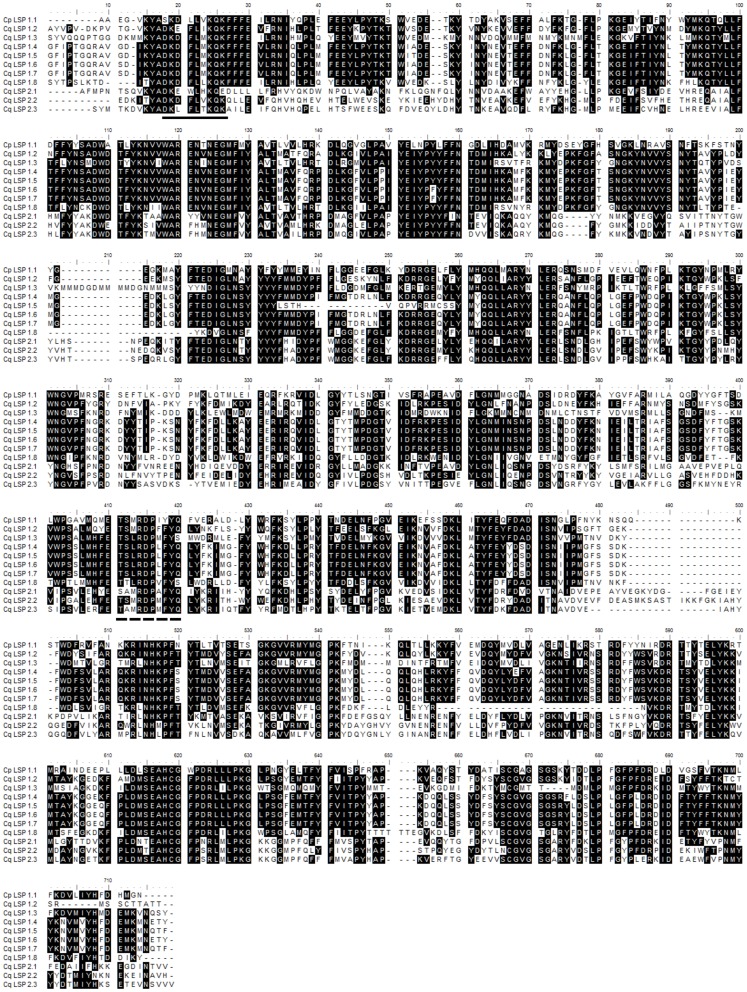
Multiple sequence alignment of the *Culex quinquefasciatus* storage proteins. The signal peptides were removed prior to the alignment. A *N*-terminal motif typical of storage proteins is underlined with a solid line; the arthropod hemocyanin pattern (T-x-x-R-D-P-x-F-Y) is underlined with a dashed line.

The alignment of the mature *Cx. quinquefasciatus* storage protein sequences (Figure 1) revealed two conserved insect storage protein motifs (ADKDFLXKQK) and (T-x-x-R-D-P-x-F-Y) located respectively at the amino terminal and a central portion of the proteins [38]–[40].

### Expression of Storage Proteins-coding Genes

**Figure 2:**
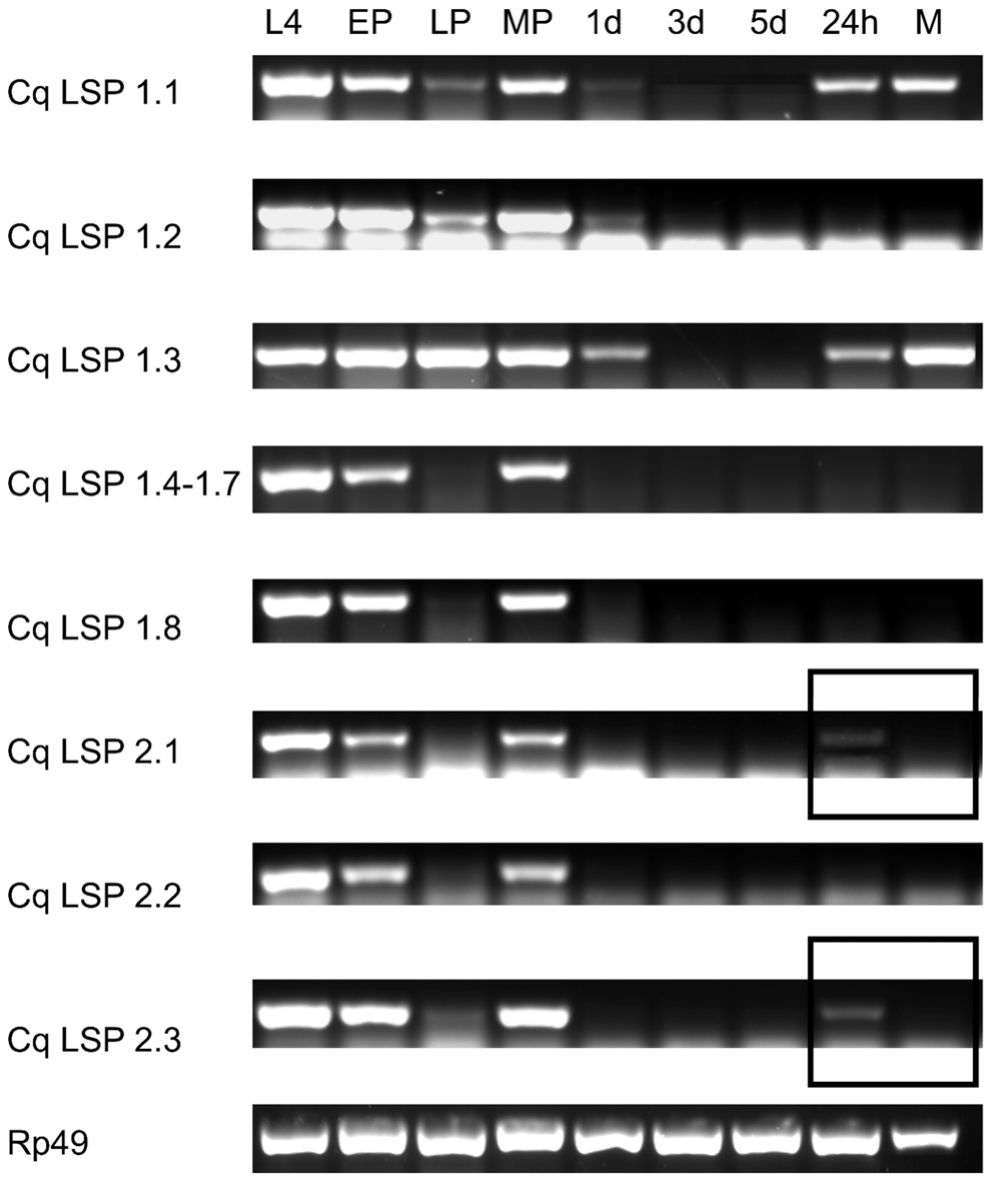
Developmental RT-PCR analysis of storage protein transcripts. Fourth instar larvae (L); early female pupae (EP, 12 hour-old); late female pupae (LP, 36 hour-old); male pupae (MP, 36 hour-old); adult females 1, 3 and 5 days after emergence (1 d, 3 d, 5 d); 7 day-old females 24 hours post blood meal (24 h) and 7 day-old adult males (M). The transcripts encoding Cq LSP 1.4, Cq LSP 1.5, Cq LSP 1.6 and Cq LSP 1.7 have highly similar sequences and the primer pair used in this study does not discriminate among them.The ribosomal protein 49 transcript (CPIJ001220-RA) was used as a constitutively expressed control.

The transcriptional profiles of the 11 *Cx*. *quinquefasciatus* storage protein encoding genes were analyzed (Figure 2). All amplicons were sequenced to verify the transcripts identities (data not show).

Fourth-instar larvae and early female pupae accumulate high quantities of storage protein encoding mRNAs. For some of the genes, low levels of transcript accumulation are still detected in 1 day-old adult females. Transcription of all eleven genes ceases in older sugar-fed females. The transcripts of Cq LSP 1.1 and Cq LSP 1.3 are present in 7 day-old adult males and blood-fed females. Cq LSP 2.1 and Cq LSP 2.3 transcription is reactivated only in blood-fed females and is not detected in 7 day-old adult males (Figure 2).

**Figure 3:**
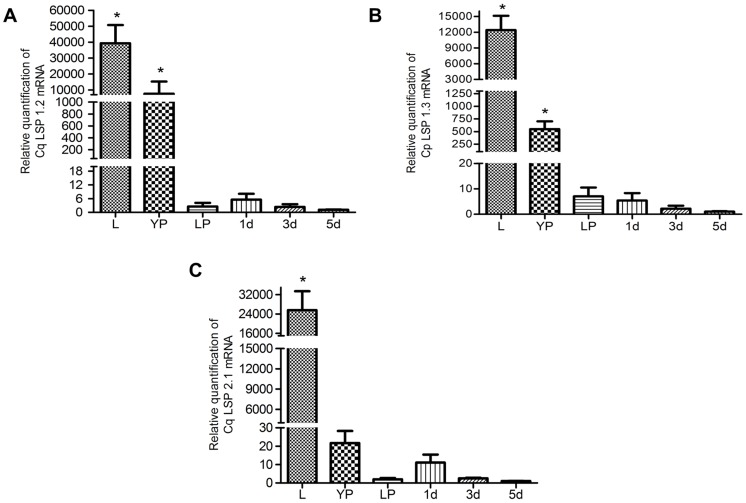
Expression profiles of storage protein transcripts during *Cx. quinquefasciatus* development. **A:** Cq LSP 1.2; **B:** Cq LSP 1.3; **C:** Cq LSP 2.1. Larvae (L); early female pupae (EP, 12 hour-old); late female pupae (LP, 36 hour-old); and adult females 1, 3 and 5 days after emergence (1 d, 3 d, 5 d), were determined by RT-qPCR. Asterisks denote qRT-PCR data with a statistically significant difference compared to the standard condition (5 d), as determined by *one*-*way* ANOVA (* = p<0.05) and Tukey’s test.

The expression profiles of Cq LSP 1.2, Cq LSP 1.3, and Cq LSP 2.1, also were analyzed by qRT-PCR (Figures 3A and 3B), validating the information described above. Cq LSP 1.2 and Cq LSP 1.3 exhibited a similar transcriptional profile: a high transcipt abundance in larvae, with a decrease in early pupae and negligible levels at the late pupa and adult stages (Figures 3A and 3B). The transcriptional profile of Cq LSP 2.1 showed mRNA abundance in larvae and a marked decrease already at the early pupal stage (Figure 3C).

### Experimental Identification of *Cx. quinquefasciatus* Storage Protein*s*

Insect storage proteins have been described as abundant components of last-instar larvae of holometabolous insects. These proteins also accumulate highly in the fat body of late larvae and early pupae. Therefore, SDS-PAGE was conducted to reveal the major larval and pupal *Cx. quinquefasciatus* hemolymph and fat body proteins.

**Figure 4:**
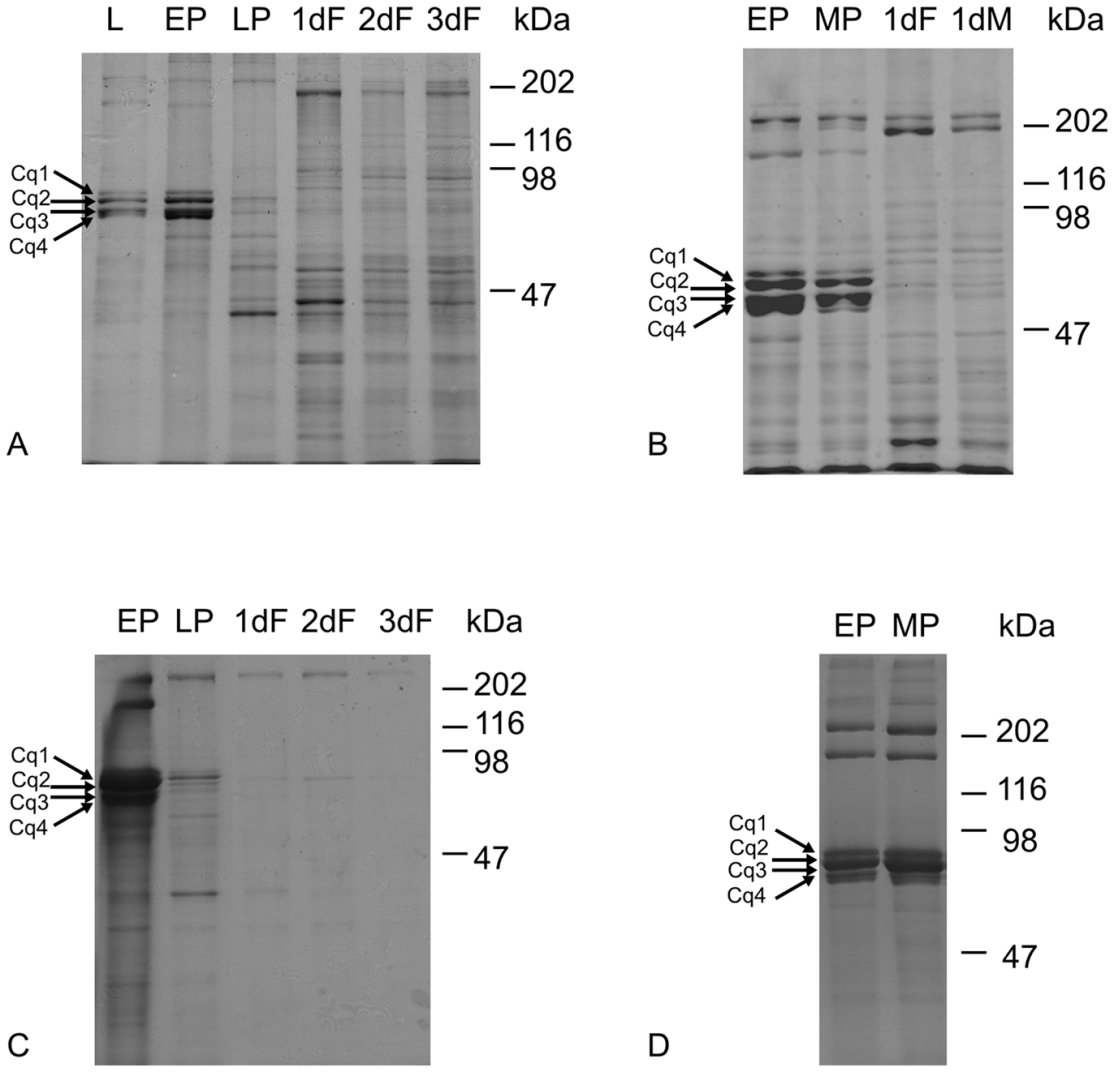
SDS-PAGE of fat body and hemolymph proteins of *Cx. quinquefasciatus*. A and B: fat body; C and D: hemolymph. Lanes represent: fourth-instar larvae (L), early female pupae (EP, 12 hour-old), late female pupae (LP, 36 hour-old), male pupae (MP, 36 hour-old), adult females 1, 2, and 3 days after emergence (1 dF, 2 dF and 3 dF), adult male 1 day after emergence (1 dM). Arrows indicate the four major protein bands, Cq1, Cq2, Cq3, and Cq4. The numbers on the right indicate the relative molecular mass in kilo Daltons (kDa).

Four bands, with apparent molecular masses of 84, 81, 74 and 72 kDa (named Cq1, Cq2, Cq3, and Cq4, respectively) were observed as the major polypeptides of late fourth-instar larval fat body extracts. Polypeptides with identical apparent molecular masses are the major components of early pupal fat body extracts (Figures 4A and 4B). A considerable lower abundance of these polypeptides was observed in the fat body of late pupae and adults (Figures 4A and 4B). Cq1, 2, 3, and 4 are abundant in the hemolymph of early pupae, declining in concentration in the hemolymph of late pupae and adults (Figures 4C and 4D).

The bands Cq1, Cq2, Cq3, and Cq4 excised from the polyacrylamide gel and whole early pupal extracts were analyzed by mass spectrometry. Cq1 corresponds to Cq LSP 2.1 and Cq LSP 2.2; Cq2 contains these same proteins, but also Cq LSP 1.2 and Cq LSP 2.3; Cq3 and Cq4 contain Cq LSP 1.2, 1.4, 1.5 and 1.6. Cq3 also contain Cq LSP 1.7 (Table 2). It is noteworthy that the proteins encoded by Cq LSP 1.1 and Cq LSP 1.3 were not identified in any of the gel fractions but were detected in the whole pupa extract. The Cq LSP 1.8 protein was not detected in any of the gel bands nor in the pupal extract.

**Table 2:** Detection of *Cx. quinquefasciatus* storage proteins by mass spectrometry.

	Gene/Protein ID	Whole pupaehomogenate	4th instar larvaehemolymph	Cq1	Cq2	Cq3	Cq4	RT-PCR
**LSP1-like**	CPIJ009032**Cq LSP 1.1**	**+**	**−**	**−**	**−**	**−**	**−**	**L P A**
	CPIJ009033**Cq LSP 1.2**	**+**	**+**	**−**	**+**	**+**	**+**	L P
	CPIJ009506**Cq LSP 1.3**	**+**	**−**	**−**	**−**	**−**	**−**	**L P A**
	CPIJ018824**Cq LSP 1.4**	**+**	**−**	**−**	**−**	**+**	**+**	L P
	CPIJ018825**Cq LSP 1.5**	**+**	**−**	**−**	**−**	**+**	**+**	L P
	CPIJ006537**Cq LSP 1.6**	**+**	**−**	**−**	**−**	**+**	**+**	L P
	CPIJ006538**Cq LSP 1.7**	**+**	**−**	**−**	**−**	**+**	**−**	L P
	CPIJ000056**Cq LSP 1.8**	**−**	**−**	**−**	**−**	**−**	**−**	L P
**Lsp2-like**	CPIJ007783**Cq LSP 2.1**	**+**	**+**	**+**	**+**	**−**	**−**	L P
	CPIJ001820**Cq LSP 2.2**	**+**	**+**	**+**	**+**	**−**	**−**	L P
	CPIJ001822Cq **LSP 2.3**	**+**	**+**	**−**	**+**	**−**	**−**	**L P A**

The bands Cq1, Cq2, Cq3, and Cq4 excised from the polyacrylamide gel (see Figure 4), and whole early pupae extracts, were analyzed by ESI mass spectrometry.

## Discussion

Holometabolous insects larvae accumulate nutrients to survive metamorphosis [41], [42]. The synthesis of storage proteins occurs predominantly in the fat body of the last-instar larva [43]. Secreted into the hemolymph, these proteins constitute the major soluble proteins of the insect at the end of the larval feeding stage [5]. During or around the time of the larval-pupal molt, protein synthesis stops and the storage proteins are endocytosed by the fat body via a specific receptor, and are stored in dense granules [5], [43].

Our *in silico* search for storage protein-encoding genes, analyses of transcript accumulation, protein electrophoresis, and mass spectrometry-based identification of larval and pupal proteins, support the occurrence of typical storage proteins in *Cx. quinquefasciatus*. Fourth-instar *Cx. quinquefasciatus* larvae and early pupae exhibit high accumulation levels of proteins ranging between 72 and 84 kDa in their fat bodies. The slight differences between the *in silico* estimated *Cx. quinquefasciatus* storage protein molecular masses and those determined by electrophoretic mobility are likely due to post-translational modifications such as glycosylation. The amount of these proteins is significantly decreased in late pupae and adult stages, consistent with their function as an amino acid reserve for metamorphosis.

In general, the accumulation of *Cx. quinquefasciatus* storage protein-encoding transcripts is in accordance with the protein content determined by electrophoresis. High transcript levels are detected in larvae [23], [44] with a sharp decline in pupal and adult stages [23]. An exception was the Cq LSP 1.8 protein that, despite having detectable levels of mRNA in larvae and pupae, could not be detected by mass spectrometry.

Differences on the expression and accumulation of storage proteins between males and females and between larval and adult stages have been previously observed. In the mosquito *Aedes altropalpus*, expression of one storage protein, AatHex-1.2, was found only in females [45], [46]. Besides AatHex-1.2, another dipteran storage protein with female-enhanced but not female-specific expression is the hexamerin-F of *Musca domestica* [8]. After a protein meal, Hex-F synthesis is strongly induced by transcription in adult female flies and to a much lesser extent in adult males. It was inferred that hexamerins can be a source of amino acids for the synthesis of vitellogenin in the fat body during the gonotrophic cycle. Here we describe two *Cx. quinquefasciatus* storage protein-coding genes (Cq LSP 2.1 and Cq LSP 2.3) that are expressed in adult females following a blood meal, and are not expressed in adult males.

In anautogenous mosquitoes, such as *Cx. quinquefasciatus* of the PIN strain used in this study, vitellogenesis is controlled hormonally by juvenile hormone (JH) and 20-hydroxy-ecdysone [47]–[51]. Vitellogenesis, initiated by the availability of amino acids that follows a blood meal, is characterized by abundant synthesis of proteins, mainly vitellogenin (Vg) that are transported via hemolymph and stored in the oocyte [52], [53]. Therefore, it is plausible to hypothesize that the expression of the genes encoding Cq LSP 2.1 and Cq LSP 2.3 is controlled by the same hormonal changes that regulate vitellogenesis. Additional studies are necessary to reveal the mechanisms that determine the sex specificity of expression of these two storage proteins in adult *Cx. quinquefasciatus*.

Currently, strategies for mosquito control are based mainly on elimination of mosquito breeding places, different formulations of chemical insecticides to kill larval and adult stages, and use of biological agents such as *Bacillus sphaericus* and *Bacillus thuringiensis* for biological control [54]. Nevertheless, the high costs of application of chemical and biologically-derived insecticides and the growing resistance developed to these agents [55]–[57] demonstrate the need of alternative strategies. Genetic manipulation of insects is becoming more and more a possible tool for their control. One of the keys for this strategy is the use of strong promoters with time, sex, and/or tissue specific expression. The *Cx. quinquefasciatus* genes encoding storage proteins identified in this work are candidates to provide strong promoters for applications in genetic manipulation of mosquitoes.

## Supporting Information

Table S1 Primers designed for detection and quantification of Cx. quinquefasciatus storage protein transcripts. (*) the primer pair designed for CPIJ001220 was used for both RT-PCR and qRT-PCR analyses. The transcripts encoding Cq LSP 1.4, Cq LSP 1.5, Cq LSP 1.6 and Cq LSP 1.7 have highly similar sequences and the primer pair used in this study does not discriminate among them. The ribosomal protein 49 transcript (CPIJ001220-RA) was used as a constitutively expressed control.
(DOCX)

Table S2 Amino acid composition of *Culex quinquefasciatus* storage proteins [percentage by frequency (mol%)].
(DOCX)
