# Supplementary material for: Culex quinquefasciatus Storage Proteins
Source: PLoS One. 2013 Oct 29;8(10):e77664. doi: 10.1371/journal.pone.0077664 (PMC3812268; doi:10.1371/journal.pone.0077664)
Supplement: Table S1 — Primers designed for detection and quantification of Cx. quinquefasciatus storage protein transcripts. (*) the primer pair designed for CPIJ001220 was used for both RT-PCR and qRT-PCR analyses. The transcripts encoding Cq LSP 1.4, Cq LSP 1.5, Cq LSP 1.6 and Cq LSP 1.7 have highly similar sequences and the primer pair used in this study does not discriminate among them. The ribosomal protein 49 transcript (CPIJ001220-RA) was used as a constitutively expressed control. (DOCX) [file pone.0077664.s001.docx]

**Table S1. Primers designed for detection and quantification of Cx. quinquefasciatus storage protein transcripts.**

| Gene ID | VectorBase accession number | Oligonucleotides |
| --- | --- | --- |
| Cq LSP 1.1 | CPIJ009032 | F- 5’ GCCGGGTTATTTTCACTCAT 3’  R- 5’ CATCGACTTTGAAAATGTTGAGTT 3’ |
| Cq LSP 1.2 | CPIJ009033 | F- 5’ CGGTGTTGGATCTGGCTCCA 3’ (qRT-PCR)  R- 5’ GTCGCTGTGGTACACGATGA 3’ ( qRT-PCR )  F- 5’ CACTACCGACCGATTGATGA 3’  R- 5’ TGTTTTGGTTGTCAATTGATTTT 3’ |
| Cq LSP 1.3 | CPIJ009506 | F- 5’ GGCTTCCCCTTCGACCGGAA 3’ (qRT-PCR )  R- 5’ TTGGTTGACCTTCATTTCGTC 3’ ( qRT-PCR )  F- 5’ TTTAATCACACTAAGCCGTTCG 3’  R- 5’ TGAGCAAGCAACTCTCCATT 3’ |
| Cq LSP 1.4  Cq LSP 1.5  Cq LSP 1.6  Cq LSP 1.7 | CPIJ018824 CPIJ018825 CPIJ006537 CPIJ006538 | F- 5’ ATAGCTTGCCGCTTGGATT 3’  R- 5’ TCGTCAAAGTGATACACCATCA 3’ |
| Cq LSP 1.8 | CPIJ000056 | F- 5’ AATAAAACGGCAACGAAACG 3’  R- 5’ TGAAGCTTTGCCATGCTTAGT 3’ |
| Cq LSP 2.1 | CPIJ007783 | F- 5’ AACCGCGAAAACTTCTACGA 3’ (qRT-PCR )  R- 5’ CGTAGAAGCTGGTGCGGTCC 3’ (qRT-PCR )  F- 5’ GATTTTCCACATTGCGTCAG 3’  R- 5’ TTTCTCTCGTTTCACTCGATCA 3’ |
| Cq LSP 2.2 | CPIJ001820 | F- 5’ TGGTTCACGCCCAATATGTA 3’  R- 5’ TTCGTTTTATTCCGACTTTATCC 3’ |
| Cq LSP 2.3 | CPIJ001822 | F- 5’ GGTTCGTGCCAAACATGTACT 3’  R- 5’ GCAGGATGCCACAACCTAAT 3’ |
| ribosomal protein 49 | CPIJ001220 | F- 5’ AGGTATCGACAACCGAGTGC 3’(*)  R- 5’ ACAATCAGCTTGCGCTTCTT 3’ (*) |

(*) the primer pair designed for CPIJ001220 was used for both RT-PCR and qRT-PCR analyses. The transcripts encoding Cq LSP 1.4, Cq LSP 1.5, Cq LSP 1.6 and Cq LSP 1.7 have highly similar sequences and the primer pair used in this study does not discriminate among them. The ribosomal protein 49 transcript (CPIJ001220-RA) was used as a constitutively expressed control.
