# Supplementary material for: Culex quinquefasciatus Storage Proteins
Source: PLoS One. 2013 Oct 29;8(10):e77664. doi: 10.1371/journal.pone.0077664 (PMC3812268; doi:10.1371/journal.pone.0077664)
Supplement: Table S2 — Amino acid composition of Culex quinquefasciatus storage proteins [percentage by frequency (mol%)]. (DOCX) [file pone.0077664.s002.docx]

**Table S2. Amino acid composition of *Culex quinquefasciatus* storage proteins** [percentage by frequency (mol%)].

| Gene ID | Cq LSP 1.1 | Cq LSP 1.2 | Cq LSP 1.3 | Cq LSP 1.4 | Cq LSP 1.5 | Cq LSP 1.6 | Cq LSP 1.7 | Cq LSP 1.8 | Cq LSP 2.1 | Cq LSP 2.2 | Cq LSP 2.3 |
| --- | --- | --- | --- | --- | --- | --- | --- | --- | --- | --- | --- |
| VectorBase accession number | CPIJ009032 | CPIJ009033 | CPIJ009506 | CPIJ018824 | CPIJ018825 | CPIJ006537 | CPIJ006538 | CPIJ000056 | CPIJ007783 | CPIJ001820 | CPIJ001822 |
| Amino acid Number | 666 | 667 | 682 | 675 | 663 | 675 | 675 | 630 | 690 | 690 | 676 |
| Molecular mass (kDa) | 78.57 | 80.42 | 83.03 | 80.95 | 79.33 | 80.96 | 80.95 | 76.27 | 82.15 | 82.24 | 80.28 |
| pI | 5.80 | 6.10 | 6.10 | 6.12 | 6.56 | 5.95 | 6.12 | 7.04 | 5.63 | 5.76 | 6.00 |
| Amino acid Compositions (mol%) | | | | | | | | | | | |
| A Ala | 4.80 | 4.05 | 1.32 | 3.11 | 3.02 | 3.11 | 3.11 | 1.27 | 4.93 | 4.64 | 4.59 |
| C Cys | 0.30 | 0.60 | 0.59 | 0.30 | 0.45 | 0.33 | 0.30 | 0.48 | 0.29 | 0.29 | 0.44 |
| D Asp | 6.31 | 6.15 | 8.65 | 6.96 | 6.79 | 7.11 | 7.11 | 8.25 | 6.52 | 6.52 | 5.62 |
| E Glu | 6.01 | 6.00 | 3.23 | 4.89 | 4.52 | 4.74 | 4.59 | 3.97 | 6.67 | 7.68 | 6.66 |
| F Phe | **8.11** | **10.49** | **7.18** | **9.93** | **9.65** | **9.93** | **10.07** | **10.16** | **7.25** | **6.52** | **8.28** |
| G Gly | 6.46 | 5.10 | 4.84 | 5.78 | 5.43 | 5.63 | 5.63 | 5.71 | 5.65 | 5.51 | 5.77 |
| H His | 1.20 | 0.75 | 1.03 | 1.04 | 1.21 | 1.04 | 1.04 | 1.27 | 2.61 | 4.35 | 3.25 |
| I Ile | 3.60 | 4.05 | 4.40 | 5.04 | 4.98 | 5.04 | 5.04 | 6.03 | 4.78 | 5.22 | 4.29 |
| K Lys | 6.31 | 8.10 | 7.04 | 7.26 | 7.09 | 7.11 | 7.11 | 7.94 | 6.52 | 6.96 | 5.77 |
| L Leu | 9.16 | 6.30 | 6.74 | 8.00 | 7.84 | 8.00 | 8.00 | 10.32 | 7.10 | 6.23 | 7.40 |
| M Met | **4.35** | **3.75** | **14.08** | **4.30** | **4.07** | **4.30** | **4.30** | **3.02** | **2.46** | **3.19** | **3.85** |
| N Asn | 5.26 | 4.35 | 5.43 | 4.74 | 4.68 | 4.74 | 4.74 | 4.60 | 4.78 | 4.20 | 4.14 |
| P Pro | 3.90 | 4.50 | 2.93 | 4.89 | 4.98 | 4.89 | 4.89 | 3.65 | 5.36 | 4.78 | 4.88 |
| Q Gln | 3.00 | 2.25 | 2.05 | 3.11 | 3.47 | 3.26 | 3.41 | 1.11 | 3.62 | 2.75 | 3.40 |
| R Arg | 4.80 | 3.45 | 4.11 | 3.85 | 3.77 | 3.85 | 3.85 | 4.13 | 4.06 | 3.48 | 4.14 |
| S Ser | 5.11 | 5.85 | 3.96 | 5.33 | 6.03 | 5.48 | 5.48 | 3.49 | 3.77 | 3.62 | 3.99 |
| T Thr | 5.71 | 5.40 | 6.16 | 5.19 | 5.43 | 5.19 | 5.19 | 7.30 | 4.06 | 4.78 | 5.03 |
| V Val | 5.41 | 5.40 | 5.57 | 4.59 | 4.98 | 4.95 | 4.59 | 5.24 | 7.25 | 7.54 | 7.69 |
| W Trp | 1.50 | 1.50 | 2.05 | 1.48 | 1.51 | 1.48 | 1.48 | 2.06 | 1.30 | 1.59 | 1.04 |
| Y Tyr | **8.71** | **11.99** | **8.65** | **10.22** | **10.11** | **10.22** | **10.07** | **10.00** | **11.01** | **10.14** | **9.76** |
